# Effects of M-Cube Wave Versus Low-Frequency Neuromuscular Electrical Stimulation on the Strength and Thickness of the Quadriceps Muscles: A Within-Subject Comparison in Healthy Young Adults

**DOI:** 10.7759/cureus.103742

**Published:** 2026-02-16

**Authors:** Shigeru Sato, Masatoshi Nakamura

**Affiliations:** 1 Department of Bio-Environmental Adaptation Sciences, Graduate School of Biomedical and Health Sciences, Hiroshima University, Hiroshima, JPN; 2 Department of Physical Therapy, Faculty of Rehabilitation Sciences, Nishikyushu University, Saga, JPN

**Keywords:** m-cube wave, muscle strength, muscle thickness, neuromuscular electrical stimulation, quadriceps

## Abstract

Background and objectives

This study compared the effects of M-cube wave and conventional low-frequency neuromuscular electrical stimulation (NMES) on the strength and thickness of quadriceps muscles in healthy young men using a within-subject design.

Materials and methods

This study included 15 untrained, healthy young adults. Each participant received two NMES protocols: an M-cube wave protocol on the quadriceps and low-frequency stimulation on the contralateral side. Sessions were performed twice per week for six weeks. Muscle strength was assessed using isometric or concentric maximal voluntary contraction (MVC-ISO and MVC-CON) torque. Muscle thickness of the rectus femoris, vastus lateralis, and vastus intermedius, and subcutaneous fat thickness were measured using ultrasonography.

Results

Both protocols significantly increased the strength (MVC-ISO: 18.9% vs. 23.3%; MVC-CON: 35.1% vs. 44.8%) and thickness of the quadriceps muscles across all examined muscles. No significant differences were observed between the two NMES waveform protocols. Both groups showed reduced thickness of the subcutaneous fat in the lateral thigh.

Conclusion

M-cube wave and conventional low-frequency NMES protocols effectively improved the strength and muscle morphology of the quadriceps muscles in healthy young men. These findings support the use of waveforms in clinical and rehabilitative settings, with waveform selection tailored to individual needs and comfort.

## Introduction

The use of neuromuscular electrical stimulation (NMES) in sports and rehabilitation settings has garnered remarkable attention owing to its potential to enhance muscle strength and hypertrophy. NMES, a technique involving the application of electrical impulses to elicit muscle contractions, has been extensively investigated as an adjunct to voluntary exercise or as a standalone intervention [1]. Although its efficacy in rehabilitation settings is well established, variations in stimulation protocols, including frequency, intensity, and electrode placement, may result in different outcomes [2]. Therefore, understanding how specific types of NMES influence muscle adaptation is critical for optimizing their use in clinical and athletic settings.

Previous studies have demonstrated the efficacy of NMES in promoting muscle strength and hypertrophy. For example, a meta-analysis by Kemmler et al. highlighted that NMES can substantially increase muscle strength and hypertrophy, particularly when combined with voluntary exercise [3]. Furthermore, other review papers have shown the positive effects of NMES on physical function, including muscle strength, muscle mass, balance, and gait function, in older adults [1], patients undergoing anterior cruciate ligament (ACL) surgery [4], and those with chronic obstructive pulmonary disease [4]. Notably, Pantović et al. compared the effects of a five-week NMES training program with a resistance training program of the same duration for students in the sports and physical education faculty [5]. Reportedly, NMES training can increase the strength of the knee extension muscle and has equal potential, if not better, than traditional resistance training exercises [5]. Akagi et al. investigated the effect of eight-week NMES training on knee extensors in healthy, untrained young males [6]. Notably, an eight-week NMES training program could increase maximal voluntary isometric contraction (MVC-ISO) of the knee extensors and induce muscle hypertrophy of the quadriceps muscles [6]. These findings underscore the potential of NMES to elicit muscle adaptations, particularly in the knee extensors, even in populations with limited voluntary muscle activity.

Although the advantages of NMES are well documented, its tendency to preferentially activate superficial muscles makes it challenging to recruit deeper muscles. Hasegawa et al. reported that a four-week NMES intervention in the early stages of ACL reconstruction significantly increased muscle thickness (MT) in the superficial muscles, specifically the vastus lateralis (VL) and rectus femoris (RF) [7]. In contrast, no considerable changes were observed in the deep muscle, vastus intermedius (VI). Akagi et al. examined the effects of an eight-week NMES intervention on knee extensor muscle volume and observed relevant hypertrophy across all targeted muscles [6]. However, their findings indicated that deep muscle VI exhibited smaller hypertrophic adaptations than superficial muscles (RF, VL, and vastus medialis [VM]) [6]. These results highlight the need to develop NMES protocols that deliver electrical stimulation to the deep muscle tissues.

Herein, we focused on NMES using Multiple Matrix Modulation waves (M-cube wave), which can simultaneously provide a relatively high frequency of 2700 Hz and a low-frequency stimulus equivalent to 4 Hz to the human body. Thus, achieving efficient stimulation of multiple and deep muscle tissues by employing an M-cube wave that applies multiple current frequencies is possible. This study investigated the effects of a six-week NMES intervention using an M-cube wave on muscle strength and MT of the knee extensors and elucidated the differences in its efficacy compared with conventional NMES protocols.

## Materials and methods

Experimental design

**Figure 1:**
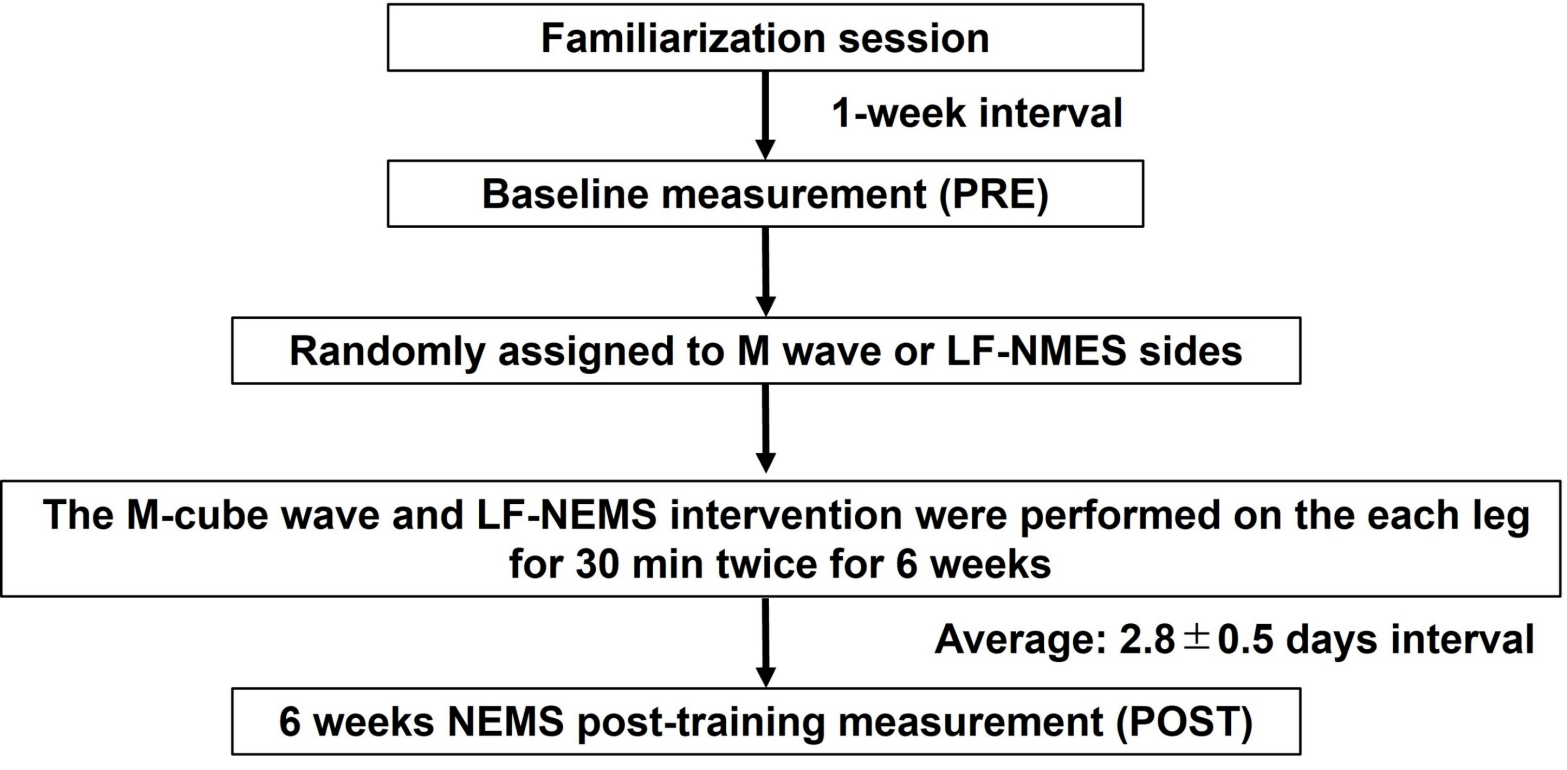
Experimental design flowchart. A familiarization session was set one week before the baseline measurements (PRE). After PRE measurements, both legs of each participant were assigned to the Multiple Matrix Modulation waves (M wave) or low-frequency neuromuscular electrical stimulation (LF-NMES) intervention sides randomly. After six weeks of NMES interventions, we performed the post-training measurements (POST).

We showed the experimental design flow chart in Figure 1. To compare the effect of the M-cube wave and low-frequency NMES (LF-NMES) on muscle strength and muscle size changes, both legs of each participant were used for the M-cube wave or LF-NMES intervention sides. The use of the dominant or nondominant leg for the M-cube wave and LF-NMES side was randomized among the participants using a random number table (Microsoft Office Excel 2007; Microsoft Corp., Redmond, WA, USA). The M-cube wave and LF-NMES intervention were performed twice for six weeks. The dependent variables comprised maximal voluntary isometric contraction (MVC-ISO) and concentric (MVC-CON) torques at 60°/s, in addition to RF, VL, and VI MT. The measurements started with ultrasound scanning for MT, followed by torque measurements for MVC-ISO and MVC-CON. Measurements were taken from the dominant leg first, followed by the nondominant leg, before (PRE) and after the six-week training (POST). A familiarization session was set one week before the baseline measurements, during which all participants performed the MVC-ISO and MVC-CON torque measurements. The post-training measurements were taken between two and four (average: 2.8 ± 0.5) days after the last NMES session.

Participants

Fifteen healthy, sedentary university students participated in this study. Participants with a history of neuromuscular disease or musculoskeletal injury involving the lower extremities were excluded. The required sample size for a repeated-measures two-way analysis of variance (ANOVA) (effect size = 0.40 [medium], α error = 0.05, and power = 0.80) using G* power 3.1 software (Heinrich Heine University, Düsseldorf, Germany) was >14 participants.

Participants were thoroughly informed about the study procedures and objectives, after which they provided written informed consent. The study was conducted in accordance with the Declaration of Helsinki and was approved by the Ethics Committee of Nishikyushu University (Procedure: 24WHN14).

Outcome assessment

Maximal Voluntary Isometric Contraction (MVC-ISO) and Maximal Voluntary Concentric Contraction (MVC-CON) Torque of Knee Extensors

The MVC-ISO torque of the knee extensors was measured at 90° knee flexion using an isokinetic dynamometer (Biodex System 3.0, Biodex Medical Systems Inc., Shirley, NY, USA). The participants sat on the dynamometer chair with an 80° hip flexion angle, with adjustable Velcro straps fixed over the trunk, pelvis, and thigh of the exercised limb. The participants were instructed to maximally contract the knee extensors for 3 s at each angle. Two repetitions with a 60-s rest between trials were performed under the PRE and POST conditions [8]. The mean of both repetitions was used for further analysis. MVC-CON torque was measured at an angular velocity of 60°/s between 0° and 90° knee flexion. From the three trials performed at PRE and POST, the highest value was analyzed [8]. Strong verbal encouragement was provided during all tests to elicit maximal effort.

Measurement of muscle thickness

B-mode ultrasonography (Venue Fit; GE Healthcare Japan, Tokyo, Japan) with an 8-MHz linear array probe was used to evaluate the MT of the quadriceps muscles. The well-trained investigator who performed the measurement has 15 years of experience in ultrasonographic measurements. Also, during ultrasonographic measurements, minimal pressure was applied to the probe to avoid tissue compression and ensure accurate assessment of muscle and subcutaneous thickness. We measured the subcutaneous fat thickness and MT of the RF, VI, and VL in participants in a supine position with a 0° hip and knee angle, similar to a previous study [9]. The VI was measured at the medial and lateral sides because of the possibility of heterogeneous architecture between the medial and lateral regions [10]. Ultrasonographic measurements were obtained at least 20 min after the participants assumed a supine position to avoid the potential for intramuscular fluid shifts from changing body position. Longitudinal ultrasound images were obtained for the VL, RF, and VI muscles, and the MT was determined as the mean of the distances between the deep and superficial aponeuroses (for VI, between the superficial aponeurosis and bone of VI) measured at both image ends [9,10]. To ensure that the same site was measured before and after the intervention, the ultrasonographic images at POST were taken while referring to the images acquired at PRE.

M-cube wave and LF-NMES intervention

M-cube wave and LF-NMES were performed on the quadriceps muscles of each leg for 30 min twice a week for six weeks. Each intervention was performed with an interval of at least 48 h. Participants were seated in a relaxed position with the stimulated leg slightly elevated and supported. Verbal guidance was provided throughout each session to ensure comfort and safety. In both M-cube wave and LF-NMES sides, two self-adhesive electrode pads (approximately 5 × 5 cm^2^) were placed over the belly of the quadriceps femoris muscle with a distance of approximately 5-7 cm between them. The electrode placement was standardized across all participants based on anatomical landmarks: one pad was positioned on the proximal portion of the RF, and the other on the superior border of the patella.

In the M-cube wave intervention side, electrical stimulation was delivered using the EXETRON 606 Plus (TechnoLink, Niigata, Japan) in C-mode (4-waves), which superimposes a low-frequency 4 Hz modulation onto a 2700 Hz carrier waveform. The stimulation was applied with a duty cycle of 4 seconds ON and 2 seconds OFF. In LF-NMES, electrical stimulation was delivered using the OMG‑S6 (TechnoLink, Niigata, Japan), a six-channel low-frequency stimulation device. The stimulation was applied at a frequency of 20 Hz, and a duty cycle of 4 seconds ON and 2 seconds OFF was applied. The intensity was gradually increased to the maximum tolerable level without causing pain, typically eliciting visible muscle contraction. This intensity was individually adjusted for each participant and reassessed at each session to maintain consistent levels of stimulation.

Statistical analysis

All statistical analyses were performed using SPSS version 29.0 (SPSS Japan Inc., Tokyo, Japan). The normal distribution of the data was confirmed using the Shapiro-Wilk test. A paired t-test was conducted between both sides to ensure the consistency of the PRE values. For all the variables, a two-way split ANOVA using two factors (test time [PRE vs. POST] and legs [M wave vs. LF-NMES legs]) was used to analyze interaction and main effects. Effect sizes (ES) are presented as partial‌ eta-squared values (ƞp2) and categorized as either small effect (ƞp2 < 0.01), medium effect (ƞp2 = 0.02-0.14), or large‌ effect (ƞp2 > 0.14) [11]. Where applicable, post hoc analyses were conducted using a paired t-test with the Bonferroni correction to evaluate the differences between PRE and POST in each condition. ES (Cohen’s d) was calculated as the difference in the mean value divided by the pooled SD between PRE and POST in each condition. ES of 0.00-0.19, 0.20-0.49, 0.50-0.79, and ≥0.80 were considered trivial, small, moderate, and large, respectively [11]. Furthermore, to investigate the relationship between changes (%) in muscle strength and thickness, Pearson’s correlation coefficient was used to compare the average changes in MVC-ISO and MVC-CON torque and the average changes in MT across all sites. The correlation strength was categorized as small (r = 0.10-0.29), moderate (r = 0.30-0.49), or large (r ≥ 0.50) [11]. A significance level of 5% was set, and all results were presented as mean ± SD.

## Results

Baseline values

Table 1 shows the characteristics of the participants. No significant differences were found between the M wave and LF-NMES legs at baseline.

**Table 1:** Baseline descriptive characteristics of the study participants The data represent means (± SD)

Age (years)	20.3±0.4
Height (cm)	166.4 ± 7.1
Weight (kg)	54.3 ± 7.2
Male: Female	7: 8
Dominant side	right: 15, Left: 0

Changes in variables after training

**Figure 2:**
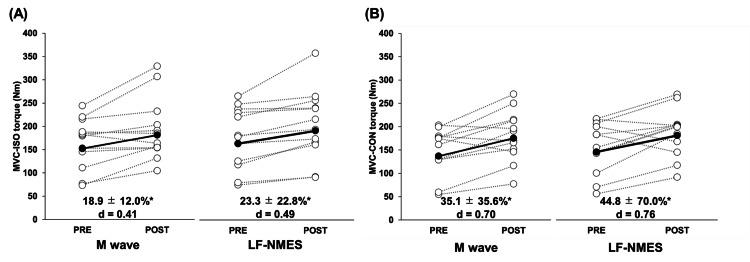
Changes in maximal voluntary isometric contraction (MVC-ISO) (A) and maximal voluntary concentric contraction (MVC-CON) (B) toques of knee extensor before (PRE) and after (POST) six weeks of neuromuscular electrical stimulation of the leg using Multiple Matrix Modulation waves (M wave) and low-frequency electrical stimulation (LF-NMES). Cohen’s d effect size is also included for the significant changes. *: significant (P < 0.05) difference from the PRE value.

Figure 2 shows the changes in the MVC-ISO and MVC-CON torques of each participant from pre- to post-training and the average (±SD) of 15 participants for the M wave and LF-NMES legs. Notable main effects of time were observed for MVC-ISO and -CON torques. However, no considerable interaction effects between time and condition were noted for any of these variables. The post hoc test revealed a significant increase in MVC-ISO (18.9 ± 12.0%, 23.3 ± 22.8%) and MVC-CON (35.1 ± 35.6%, 44.8 ± 70.0%) torque in both legs.

**Figure 3:**
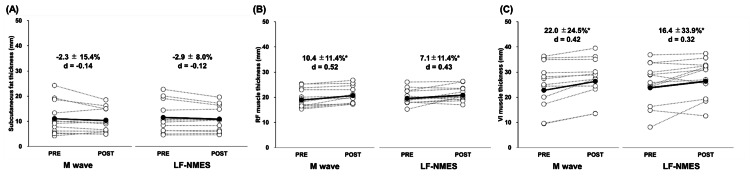
Changes in subcutaneous fat (A) and rectus femoris (RF) muscle (B) and vastus intermedius (VI) muscle (C) thickness before (PRE) and after (POST) six weeks of neuromuscular electrical stimulation of the leg using Multiple Matrix Modulation waves (M wave) and low-frequency electrical stimulation (LF-NMES). Cohen’s d effect size is also included for the significant changes. *: significant (P < 0.05) difference from the PRE value.

Figure 3 shows the changes in subcutaneous fat, MT of RF, and VI from pre- to post-training of the average (±SD) of 15 participants for the M wave and LF-NMES legs. Considerable main effects of time were observed for RF and VI subcutaneous fat, and MT. However, no notable interaction effects between time and condition (M wave vs. LF-NMES) were found for any of these variables. The post hoc test revealed a significant increase in MT of RF (M wave: 10.4 ± 11.4%, LF-NMES: 7.1 ± 11.4%) and VI (22.0 ± 24.5%, 16.4 ± 33.9%) in both legs, whereas no observable changes in subcutaneous fat were detected in either leg.

**Figure 4:**
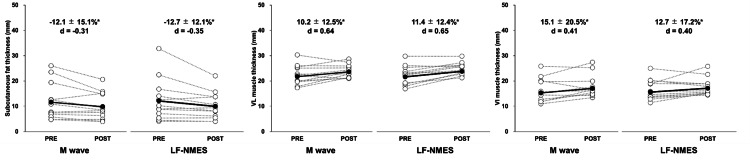
Changes in subcutaneous fat (A) and vastus lateralis (VL) muscle (B) and vastus intermedius (VI) muscle (C) thickness before (PRE) and after (POST) six weeks of neuromuscular electrical stimulation of the leg using Multiple Matrix Modulation waves (M wave) and low-frequency electrical stimulation (LF-NMES). Cohen’s d effect size is also included for the significant changes. *: significant (P < 0.05) difference from the PRE value.

Figure 4 depicts the changes in subcutaneous fat and MT of VL and VI from pre- to post-training of the average (±SD) of 15 participants for the M wave and LF-NMES legs. The main effects of time were observed for subcutaneous fat and MT of VL and VI. However, no notable interaction effects between time and condition were found for any of these variables. The post hoc test revealed a significant decrease in subcutaneous fat (−12.1 ± 15.1%, −12.7 ± 12.1%) and an increase in MT of VL (10.2 ± 12.5%, 11.4 ± 12.4%) and VI (15.1 ± 20.5%, 12.7 ± 17.2%) in both legs.

Pearson’s correlation coefficient indicated a significant moderate positive correlation between the average change in muscle strength and the average change in MT (r = 0.441, p = 0.015).

## Discussion

This study compared the effects of M wave and conventional LF-NMES on the adaptation of the quadriceps muscles in healthy young men. Both protocols similarly increased MVC-ISO and MVC-CON torque, in addition to the RF, VL, and VI MT. In addition, both conditions resulted in comparable reductions in the subcutaneous fat in the lateral thigh region. Although these findings did not completely support our initial hypothesis that the M-cube wave would produce superior adaptations, both NMES modalities can induce remarkable improvements in muscle strength and hypertrophy over a relatively short training period.

The M wave and conventional LF-NMES protocols resulted in comparable improvements in knee extensor strength, with increases of 18.9% and 23.3% in MVC-ISO torque and 35.1% and 44.8% in MVC-CON torque, respectively. These muscle strength gains were relatively larger than those reported previously [5,6]. For instance, Akagi et al. reported a 9.3% increase in MVC-ISO torque following an eight-week NMES intervention consisting of 13-min sessions, three times per week [6]. Similarly, Pantović et al. demonstrated a 10.6% increase in MVC-CON torque after a five-week NMES protocol (18 min per session, three sessions per week). Notably, the strength gains were comparable with those achieved using conventional resistance training [5]. Although direct comparisons across studies are limited owing to methodological differences, such as stimulation duration, intervention period, and waveform characteristics [12], the relatively greater strength improvements observed herein may reflect the use of higher stimulation intensities and the inclusion of untrained participants, who are known to exhibit more pronounced neural adaptations. During the initial stages of strength development, such gains are primarily attributed to enhanced motor unit recruitment, increased firing frequency, and improved neuromuscular coordination [13]. Neural adaptation plays a notable role in increasing muscle strength achieved through NMES protocols [14,15]. An increase in MT was observed concomitantly with an increase in muscle strength. Furthermore, the present findings revealed a significant correlation between changes in MT and the increase in muscle strength (r = 0.441, p = 0.015), corroborating Akagi et al.’s findings [6], which suggest that muscle hypertrophy contributes to the increase in muscle strength. Therefore, the high-intensity NMES protocols employed in both conditions were sufficient to elicit meaningful strength adaptations over the six-week training period.

The M wave and LF-NMES protocols substantially increased the RF, VL, and VI MT. This finding contrasts with that of Hasegawa et al. [7], who reported no considerable change in VI thickness following a four-week NMES program. In contrast, Akagi et al. observed an increase in the overall quadriceps muscle volume after an eight-week NMES intervention using belt electrodes; however, the effect size for VI hypertrophy was small (d = 0.167), indicating a limited response in the deep musculature [6]. Notably, our study assessed MT using ultrasound, whereas Akagi et al. employed magnetic resonance imaging to evaluate muscle volume, limiting the direct comparability between the two approaches [6]. Nonetheless, the findings of this study suggest that regardless of waveform characteristics, NMES protocols ensuring sufficient stimulation intensity can induce meaningful morphological adaptations even in deep muscles such as the VI. Future studies should explore optimal stimulation conditions enhancing current penetration into deeper muscle tissues, particularly in clinical populations with neuromuscular impairment.

Notably, both NMES protocols led to a considerable reduction in subcutaneous fat thickness in the lateral thigh region. However, strong evidence demonstrating consistent and clinically meaningful reduction in subcutaneous fat through NMES alone remains limited. NMES alone is unlikely to achieve substantial reductions in body fat without additional interventions such as dietary control, resistance training, and aerobic exercise [16]. Similarly, Hioki et al. observed that echo intensity, serving as a proxy indicator of intramuscular fat content, did not improve after a 12-week NMES training regimen in older adults [17]. Dietary intake was not regulated in our study; therefore, the observed site-specific reduction in subcutaneous fat in the lateral region was unlikely to represent a direct effect of NMES. In conclusion, short-term NMES protocols, regardless of waveform characteristics, are unlikely to considerably reduce the subcutaneous or intramuscular fat content.

Overall, the study findings demonstrated that the M wave and conventional LF-NMES protocols effectively increased the thickness and strength of the quadriceps muscles in healthy young men. NMES is widely recognized as a time-efficient and joint-sparing method for enhancing muscle strength, particularly in individuals who cannot engage in conventional resistance training because of injury, surgery, and/or other limitations [18,19]. Given that both waveforms produce comparable neuromuscular adaptations, waveform selection can be based on patient comfort, device availability, and clinical context. However, NMES should be viewed as a complementary tool rather than a replacement for traditional exercises. Future studies should investigate the effects of individualized NMES protocols, long-term adaptations, and potential synergistic benefits of combining NMES with resistance or aerobic exercise. Such efforts will help optimize the practical application of NMES across diverse settings and populations.

This study had several limitations. First, the sample was limited to healthy, untrained young adults, which restricts the generalizability of the findings to other populations, such as trained individuals, older adults, clinical populations, and those with neuromuscular impairments. The inclusion of untrained participants may have contributed to the relatively large increase in muscle strength. Second, the intervention period was relatively short, and the long-term effects of NMES on muscle hypertrophy, strength gain, and fat reduction remain unclear. Third, subjective parameters, such as discomfort during stimulation, perceived exertion, and pain, were not evaluated. Although all participants completed the intervention with 100% adherence, these factors may have affected the acceptability and compliance with NMES protocols in clinical settings. Fourth, because this study employed a within-subject design, wherein each leg received a different NMES intervention, the potential influence of cross-education effects cannot be ruled out. Unilateral neuromuscular stimulation or training may induce contralateral strength gain via neural adaptations [20,21]. Therefore, some crossover effect may have contributed to the strength improvements observed in both legs. Finally, dietary intake and physical activity levels were not strictly controlled during the study period. These uncontrolled variables may have influenced changes in the thickness and size of subcutaneous fat.

## Conclusions

The M wave and conventional LF-NMES protocols yielded similar enhancements in the strength and thickness of the quadriceps muscles in healthy, untrained young adults after a six-week intervention period. Reportedly, both M cube wave and LF-NMES intervention can induce neuromuscular adaptations, including those in deep muscles such as the VI, when adequate stimulation intensity is maintained. Future research should investigate the effects of NMES in more diverse populations, explore long-term adaptations, and determine the optimal parameters for integrating NMES into comprehensive rehabilitation or performance-enhancement programs.
